# *In Vivo* Noninvasive Detection of Brown Adipose Tissue through Intermolecular Zero-Quantum MRI

**DOI:** 10.1371/journal.pone.0074206

**Published:** 2013-09-10

**Authors:** Rosa T. Branca, Le Zhang, Warren S. Warren, Edward Auerbach, Arjun Khanna, Simone Degan, Kamil Ugurbil, Robert Maronpot

**Affiliations:** 1 Department of Physics and Astronomy, University of North Carolina at Chapel Hill, Chapel Hill, North Carolina, United States of America; 2 Biomedical Research Imaging Center, University of North Carolina at Chapel Hill, Chapel Hill, North Carolina, United States of America; 3 Applied Science and Engineering, University of North Carolina at Chapel Hill, Chapel Hill, North Carolina, United States of America; 4 Department of Chemistry, Duke University, Durham, North Carolina, United States of America; 5 Center for Magnetic Resonance Research, University of Minnesota, Minneapolis, Minnesota, United States of America; 6 Experimental Pathology Laboratories, Inc., Research Triangle Park, North Carolina, United States of America; University of Bonn, Germany

## Abstract

The recent discovery of active Brown Adipose Tissue (BAT) in adult humans has opened new avenues for obesity research and treatment, as reduced BAT activity seem to be implicated in human energy imbalance, diabetes, and hypertension. However, clinical applications are currently limited by the lack of non-invasive tools for measuring mass and function of this tissue in humans. Here we present a new magnetic resonance imaging method based on the normally invisible intermolecular multiple-quantum coherence ^1^H MR signal. This method, which doesn’t require special hardware modifications, can be used to overcome partial volume effect, the major limitation of MR-based approaches that are currently being investigated for the detection of BAT in humans. With this method we can exploit the characteristic cellular structure of BAT to selectively image it, even when (as in humans) it is intimately mixed with other tissues. We demonstrate and validate this method in mice using PET scans and histology. We compare this methodology with conventional ^1^H MR fat fraction methods. Finally, we investigate its feasibility for the detection of BAT in humans.

## Introduction

Obesity is rapidly spreading across most developed countries and is thought to be more harmful to health than alcohol or smoking because of its association with many other medical conditions [1]. At a fundamental level, obesity is the result of an imbalance between energy intake and energy expenditure. The latter is very difficult to quantify and recent work suggests that it can be altered by the function of Brown Adipose Tissue (BAT) [2]. BAT [3], [4] is a type of fat that modulates both basal (cold exposure) and inducible (overeating related) energy expenditure in mammals, thereby affecting whole-body metabolism and modifying susceptibility to weight gain [5]–[8]. It is considered to be the “good fat” that, unlike the white “bad fat”, burns calories to produce heat through a process called non-shivering thermogenesis.

While in small animals malfunction of this tissue is known to cause obesity, in humans the role and the incidence of tissue is less clear. In fact until recently, BAT was thought to exist in humans only in infancy and early childhood.[9] However, combined ^18^F-fluorodeoxyglucose positron emission tomography (^18^F-FDG-PET) and computed tomography (CT) scans have identified active BAT in adults and shown a strong correlation between BAT activity and the basal metabolic rate [3], [10]–[12]. As it turned out, BAT was missed in adult humans because of its diffuse anatomical distribution: this tissue is present only in scattered amounts in the neck and chest areas, around major blood vessels [13], muscles [14] or white fat [15]. Nevertheless, it is estimated that BAT activity could account for up to 20% of daily energy expenditure in an adult human [16].

Although this tissue is a clear target for obesity treatments, the current modality of choice for imaging metabolically active BAT in humans, PET/CT, presents significant limitations. BAT metabolism relies on fatty acid consumption, not glucose consumption, so ^18^F-FDG-PET is highly nonspecific. Moreover, confounding factors such as blood glucose levels and room temperature conditions [11], [17], [18] may affect glucose uptake in BAT. Other, more specific, PET metabolic tracers have been used to estimate BAT oxidative capacity and fatty acid uptake [19] during thermogenic activity in humans. Still, radiation exposure from PET imaging, although considerably smaller than PET/CT imaging [20], precludes the repetitive BAT screening in healthy and young subjects needed to determine the physiological relevance of BAT in humans [21], [22].

MRI is very attractive for BAT studies since it is non-invasive, does not deliver mutagenic radiation, and has no limitations in imaging penetration depth. More interestingly, the difference in chemical shift between water protons and fat protons makes it possible to differentiate lean from fatty tissues as well as normal white fat (WAT) from brown fat. While brown fat is characterized by multilocular brown adipocytes with average water content of about 50%, white fat is characterized by unilocular adipocytes with water content of less than 10%. As a consequence, fat fraction measurements performed with ^1^H MR can be used to differentiate these two tissues, at least in rodents [23]–[27]. However, in adult humans this tissue is present only in scattered amounts, and partial volume effects caused by different types of cells in a single voxel (for example WAT, BAT, and muscle) lead to both false positive and false negative findings. Although, in principle, MR resolution could easily be increased to reduce partial volume effects, in practice this is unfeasible. For example, an isotropic increase in image resolution by a factor of two (i.e. a 8-fold reduction in pixel volume) results in a factor of 8 loss in SNR, which can be compensated only by a 64-fold increase in acquisition time.

**Figure 1:**
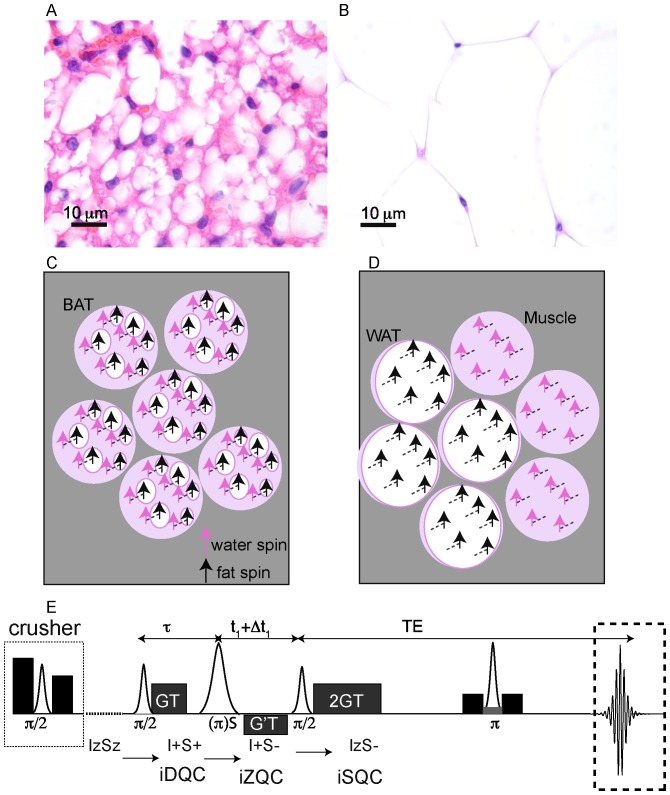
Origin of the water-fat iZQC signal from BAT (BATSCI). (**A–B**) Histological haematoxylin and eosin (H&E) staining of BAT (**A**) and white fat (**B**) from a mouse showing different cell morphologies. Brown fat cells present multiple smaller lipid vacuoles and higher hydration level and are usually smaller than white fat cells, which are made by a single large lipid droplet. (**C**) Cartoon showing the different cellular structures and the origin of the iZQC signal in BAT: unlike in white fat, in BAT water and fat spins are mixed together at the cellular level such that the selection of a small correlation distance can select the BATSCI signal only from BAT (**D**) Scheme of the radio frequency pulse sequence used in the experiments for the detection of BAT.

In this paper we show that we can overcome these weaknesses of conventional MRI by using the non-linear MR signal generated by intermolecular zero-quantum coherences (iZQCs) that originates from closely spaced water and fat spins [28]. These coherences are generated by simultaneous and opposite transitions of water and fat spins separated by a user-controlled “correlation distance”, a distance defined as half the modulation created in the nuclear magnetization by the applied pulsed field gradients: dc = 1/(2γGT), typically 20–100 µm. A critical feature is that the correlation distance can be much finer than the image resolution, allowing us to probe structure at a much smaller scale [29], without loss of sensitivity [30]. More specifically, by choosing a correlation distance much smaller than the image resolution and comparable to the cellular size, we can suppress coherences between water and fat spins that reside in different tissues within an image voxel (WAT and muscle, for example), while retaining and enhancing coherences between water and fat spins that reside in the same cells or tissue (BAT). This is briefly outlined in figure 1.

Building upon our previous spectroscopy studies in rodents [28], where we found a strong association between the mass of brown adipose tissue and the intensity of the iZQC signal from closely spaced water and fat spins, here we demonstrate and validate, with both histology and ^18^FDG-PET scanning, that this signal does indeed originate from brown adipose tissue and can therefore be used to specifically detect and map this tissue in rodents. Finally, we investigate the feasibility to use this signal, which we call BATSCI (BAT-Specific Coherence Imaging), to detect human BAT, using both *in vitro* and *in vivo* experiments.

## Results

### Phantom Results

Figure 1.D shows the sequence used to acquire the BATSCI signal. In this sequence, as described in [28], the BATSCI signal coming from water-fat iZQCs evolves first as a water-fat double quantum coherence (iDQC) during the delay tau, as zero quantum coherence during the second evolution delay t1, and finally as single quantum coherence during the acquisition time. This specific coherence pathway is selected by a GT/2 GT gradient combination that, during the acquisition time, allows us to refocus only single quantum coherences that have evolved as double quantum coherences during the tau delay. The extra gradient pulse G’T, on the other hand, selects the evolution of the water-fat iZQC signal during the t1 delay and suppresses both homomolecular (water-water and fat-fat) coherences and heteromolecular water-fat iDQCs.

This sequence is used to collect 2D iZQC spectra as well as to map the BATSCI signal in both animals and humans. BATSCI maps are collected by a CSI type acquisition scheme. Briefly, a series of 2D iZQC images are acquired with a different iZQC evolution times, t1. A Fourier transform along the t1 dimension allows us to display the iZQC spectrum from which a BATSCI map can be obtained.

**Figure 2:**
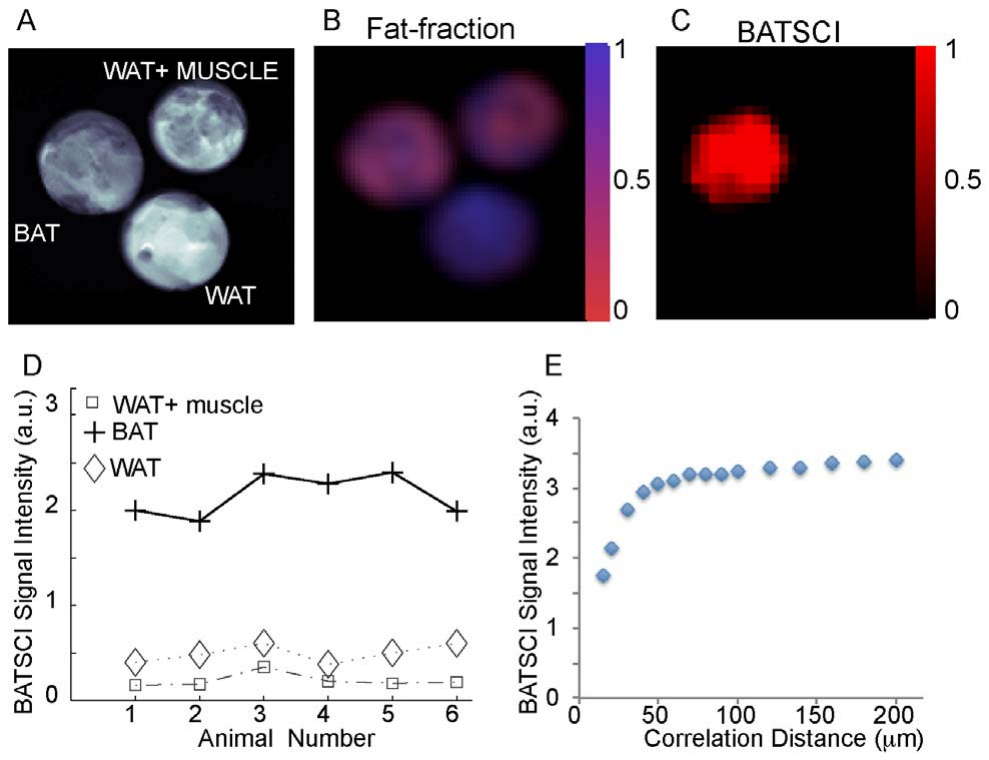
In vitro detection of excised mouse BAT. (**A**) Axial image of three samples containing BAT, WAT, and a mixture of WAT and muscle. (**B**) Fat fraction as measured by a conventional CSI sequence showing similar water content for the BAT and for the mixed sample. (**C**) BATSCI image showing the presence of a strong signal only in the BAT sample. (**D**) BATSCI signal normalized to the nearby fat-fat iZQC signal from different tissues and from different mice. (**E**) BATSCI signal intensity as function of the correlation distance as obtained from a phantom containing excised mouse BAT.

In figure 2 we compare the performance of this sequence to the more established MR fat fraction measurement method for the detection of excised BAT [23], [31]. While the fat-fraction measurement clearly differentiates samples made by 100% BAT from samples made by 100% WAT, it does not distinguish BAT from the WAT/muscle mixture. At the relative coarse resolution (0.72 mm^3^) used in this experiment, which is still higher than the resolution typically used for human brown fat MRI studies (∼1 mm^3^), the BAT and the WAT/muscle mixture look exactly the same since they have a similar water/fat faction (2.B). On the other hand, the water-fat iZQC signal for these samples is very different (2.C). The water-fat iZQC signal was present only in the BAT sample, while it was close to the noise level in the WAT-muscle mixture, despite the similar water-fat content of these two samples and the much lower resolution used for this imaging experiment (8 mm^3^). This is because the intensity of the water-fat iZQC signal depends not only on the relative concentration of water and fat spins, but also on their relative distribution over a distance smaller or equal to the selected correlation distance. The correlation distance for these experiments was selected to be ∼80 µm, much smaller than the nominal image resolution, but big enough to allow the detection of BAT of different sizes. At this scale, water and fat spins are still mixed together in the BAT sample (giving a BATSCI signal), while in the WAT/muscle mixture they are not since they are part of different tissues.

Figure 2.D shows the intensity of BATSCI signal normalized to the intensity of the nearby fat-fat iZQC signal obtained from BAT, WAT, and WAT+muscle (∼50/50 weight ratio) tissues excised from 6 different animals (C57 lean mice, 6 weeks old). Unlike the WAT and the WAT+muscle mixture, the BAT clearly presents a high BATSCI signal, which in most cases is more than ten times higher than that found for the other two tissues.

The dependence of the BATSCI signal intensity on the correlation distance is shown in Figure 2.E for an excised tissue sample of interscapular mouse BAT. As the graph shows, the optimal correlation distance is between 50 µm and 100****µm. As expected, lower values lead to a strong diffusion weighting and to a reduction of the BATSCI signal intensity, while higher values, though seemingly resulting in a slightly higher BATSCI signal, lead to a contamination from other coherences that may not be completely dephased by the correlation gradients.

### *In vivo* Mapping of BAT and Correlation with ^18^F-FDG-PET Scans and Fat Fraction Measurements

**Figure 3:**
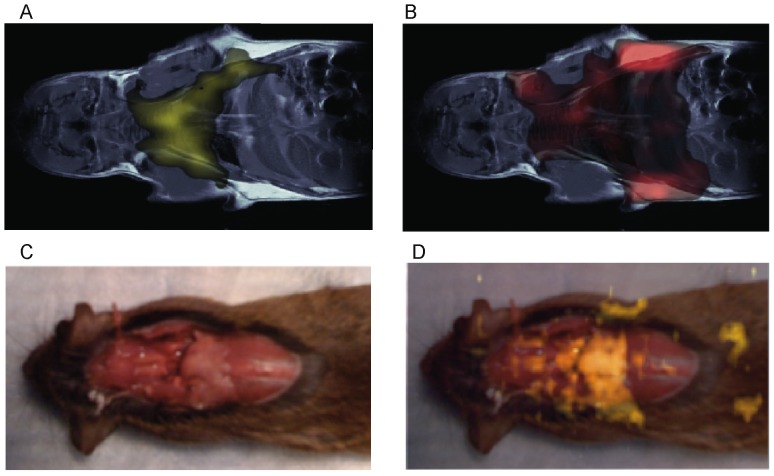
In vivo mapping of BAT using the BATSCI method. (**A**) BATSCI map in a C57 mouse highlighting the BAT depot. (**B**) Fat-fat iZQC map on the same C57 mouse highlighting both WAT and BAT depots. (**C**) Photograph of a Caveolin-1 null mouse showing the interscapular brown fat depot (pale pink). (**D**) BATSCI map overlapped on the anatomical photograph, highlighting the interscapular BAT depot and the peri-renal BAT depots (not visible in the photograph).

*In vivo* imaging studies on several mouse strains (4-Balb/c, 6-C57, 2-nude mice, 2-caveolin-1 nul mice) also show the possibility to map BAT *in vivo* in the mouse body. In figure 3 we show typical water-fat iZQC (BATSCI) maps (3.A) and fat-fat iZQC maps (3.B) obtained from a 2 month old C57 mouse. While the fat-fat iZQC signal simply highlights fatty tissues (both BAT and WAT), the BATSCI signal specifically highlights the butterfly shaped BAT depots, demonstrating that this signal can clearly be used to specifically detect the BAT depots in the body.

In figure 3.D we show the same experiment on one of the caveolin-1 nul mouse (Cav-1 nul). In this strain the interscapular brown fat (often unnoticeable in the wild strain) protrudes out of the overlying scapula and can easily be identified in the post-mortem photograph (Fig. 3.C). This depot along with the peri-renal BAT depot (not visible in the photograph), which in this mouse strain still contains abundant BAT, is clearly identified as BAT by the water-fat iZQC signal.

**Figure 4:**
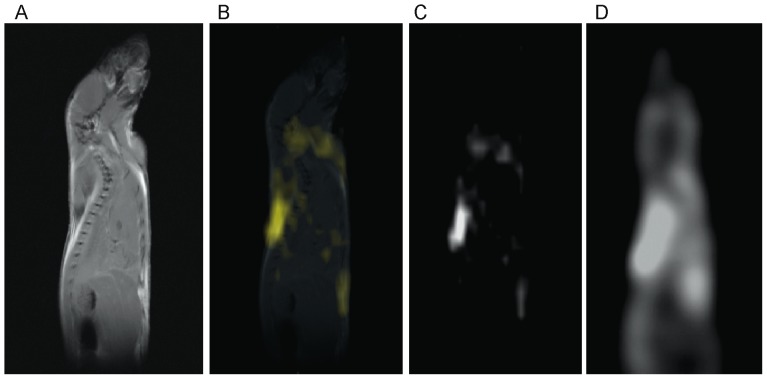
Correlation between BATSCI maps and the BAT activity maps obtained with ^18^F-FDG-PET. (**A**) Anatomical sagittal spin echo MR image of a young C57 mouse. (**B**) BATSCI map of the same mouse overlapped to the anatomical image. (**C**) BATSCI map showing the interscapular brown fat area as well other BAT depots near the neck (**D**) BAT activity map obtained by ^18^F-FDG-PET of the same mouse acquired after BAT stimulation. ^18^F-FDG-PET scans show active BAT as well as other metabolically active (heart and brain) tissues, but not inactive BAT.

In figure 4 a standard, high resolution, sagittal MRI spin-echo image on a 4-week old C57 mouse, along with the corresponding low resolution BATSCI map and the BAT activity map obtained on the same animal with ^18^F-FDG-PET scans are presented. As expected from previous studies [32], the PET scan reveals intense ^18^F-FDG uptake in BAT, in the Harderian glands and in the myocardium. ^18^F-FDG uptake in BAT is lower than in the myocardium and higher than in brain (below the image threshold). The BATSCI map reveals a strong signal only from areas corresponding to BAT: the interscapular, the axillary, and the cervical area, further confirming the suitability of this method to specifically detect BAT.

**Figure 5:**
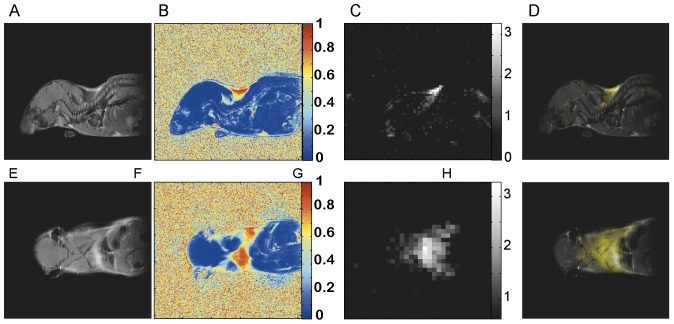
Comparison between BATSCI maps and fat fraction maps for the detection of BAT. (**A**) Anatomical sagittal spin echo MR image of a young C57 mouse. (**B**) Fat fraction map of the same animal. (**C**) BATSCI map showing the interscapular brown fat depot. (**D**) BATSCI map overlaid on spin-echo image, illustrating the intrascapular BAT depot. (**E**) Anatomical coronal spin echo MR image of the same animal. (**F**) Coronal Fat fraction map of the same animal. (**G**) BATSCI map showing the interscapular brown fat area. (**H**) BATSCI map overlaid on spin-echo image, illustrating the intrascapular BAT depot.

In figure 5 BATSCI is compared to relatively higher resolution (234 µm×156 µm in plane resolution, 2 mm slice thickness) fat fraction measurements in a C57 mouse. Fat fraction measurements clearly show the interscapular BAT depot, which presents a fat fraction ranging between 80% and 30%. The BATSCI method, on the other hand, provides background free maps of the same BAT depots, despite the use of a much coarser resolution (1.8 mm×1.25 mm in plane resolution, 40 mm slice thickness).

### *In vivo* Detection of BAT Activity

The ability of BATSCI to detect and distinguish active and inactive BAT mass is an important advantage as compared to PET. We demonstrate this with a series of BATSCI experiments before, during, and post BAT stimulation by norepinephrine (NE), a catecholamine with predominant alpha-1 and some beta-1 adrenergic stimulation.

**Figure 6:**
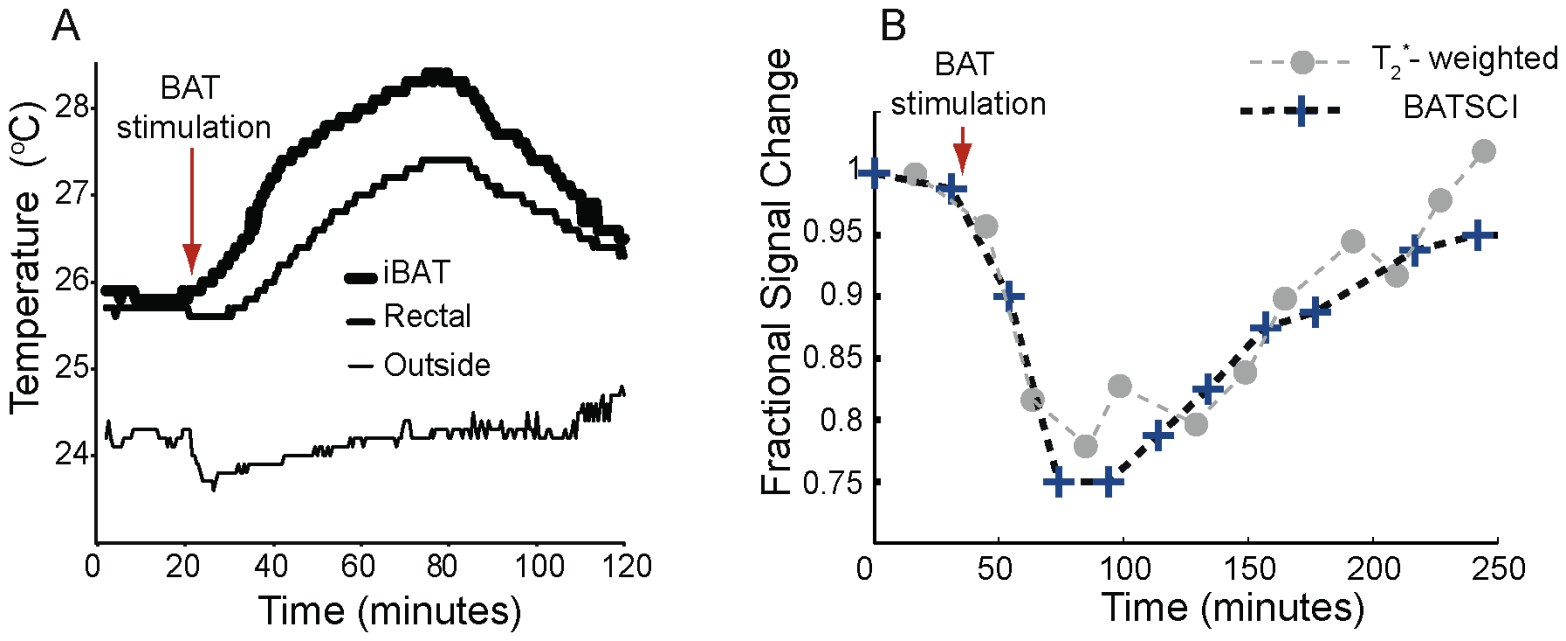
Detection of BAT activity with the BATSCI signal. (**A**) Temperature behavior of BAT in a healthy balb/c mouse after stimulation by NE injection showing the effect of the drug. After the injection of the BAT stimulant BAT temperature rises followed by rectal temperature (**B**) BATSCI signal intensity and T2* -weighted signal intensity from the interscapular BAT as function of time, before during and after stimulation of BAT by NE.

Injection of NE has largely been used in rodents to stimulate BAT thermogenesis [4], [33]. After an intraperitoneal (I.P.) injection of this drug, BAT metabolic activity rapidly increases and remains high for several minutes. During BAT activity, fatty acids are rapidly oxidized and converted into heat. This results in a local increase in BAT temperature, followed by an increase in the overall body temperature that can easily be measured in small rodents using thermocouples (Figure 6.A). Since the oxidation of fatty acid requires oxygen, oxygen consumption during BAT thermogenesis increases more than tenfold. Because this increase is not compensated by an adequate increase in blood flow (oxygen is almost completely extracted from blood flowing through BAT [34], [35], as it occurs in brain during neural stimulation, blood oxygenation in BAT veins drops below normal levels [35]. This leads to a local increase in deoxyhemoglobin (a paramagnetic agent that inherently shortens the spin transverse relaxation time) and to a drop of the standard MR signal that persists through the entire BAT activity, as we have already demonstrated [36]. This signal drop can be used to detect BAT activity in real time as it is shown in Figure 6.B. The local increase in deoxyhemoglobin also reduces the BATSCI signal intensity. However, since the BATSCI signal is insensitive to macroscopic susceptibility variations [30], for example from respiratory motion, the intensity pattern of the BATSCI signal more closely follows BAT activity. As can be seen in Figure 6.B, after the injection of BAT stimulant, while the conventional T^2*^ weighted signal decreases by 22%, the BATSCI signal decreases by almost 25%, as the result of broadening of the BATSCI resonance line due to a microscopic increase in magnetic susceptibility. This reduction lasts during the entire BAT activity followed by a slow signal recovery as the efficacy of norepinephrine on BAT starts to diminish. On the other hand no appreciable signal variation was noticed in the nearby muscle and white fat, consistently with what we have previously observed [36].

### *In vitro* Detection of Human Brown Adipose Tissue

We have also used this method to examine the presence of BAT in fatty tissues excised from 11 human autopsy cases of both sexes, aged from 28 to 95 years old, using the small animal imaging scanner. For each subject, fatty tissues were excised from both the abdominal and the supraclavicular areas. All samples were then analyzed with conventional NMR spectroscopy methods, to evaluate their overall fat fraction, and with BATSCI, to detect their overall BAT content. To determine the % of adipose tissue containing brown fat cells in the tissues analyzed we then used immunohistochemical staining for UCP1. UCP1 is a marker gene for brown adipose tissue. It plays a crucial role for BAT thermogenesis since it uncouples ATP synthesis from electron transport so that energy is dissipated to produce heat. The presence of this protein is therefore essential for the identification of a fat depot as BAT depot [4], [37].

**Figure 7:**
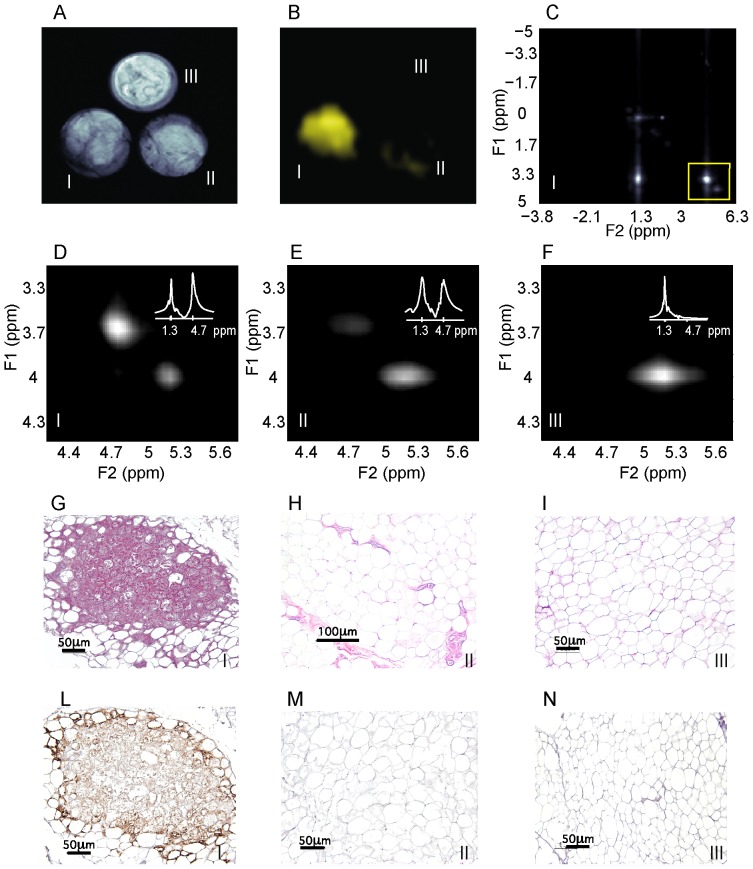
In vitro detection of human BAT using the BATSCI signal. (**A**) Spin echo image of 3 human BAT samples: supraclavicular fat from a 27 year old male (I, bottom left), supraclavicular fat from a 95 year old female (II, bottom right) and abdominal fat from the same 95 year old female (III, top) (**B**) BATSCI map obtained from the same 3 samples. Only the fat sample excised from the supraclavicular area of the 27 years old male (bottom left) shows a significant BATSCI signal. (**C**) 2D iZQC spectrum acquired on the 27 year old male supraclavicular fat sample highlighting (yellow box) the region containing the water-fat and the fat-fat iZQC peak. (**D**) 2D-iZQC spectrum showing the water-fat (BATSCI) and the fat-fat iZQC peak from the supraclavicular fat sample of the 27 year old male, along with the ^1^H-MRS spectrum (inset). (**E**) 2D-iZQC spectrum from the supraclavicular fat of the 95 year old female showing a predominant fat-fat iZQC peak, along with the ^1^H-MRS spectrum (inset). (**F**) iZQC spectrum from the abdominal area of the 25 year old female showing only a fat-fat iZQC peak, along with the ^1^H-MRS spectrum (inset). (**G–I**) Histological haematoxylin and eosin (H&E) staining of BAT from the 27 year old supraclavicular fat (**G**), the 95 year old supraclavicular fat (**H**) and 95 year old abdominal fat (**I**). (**L–N**) immunohistochemistry of the same samples to detect UCP1 expression.

**Figure 8:**
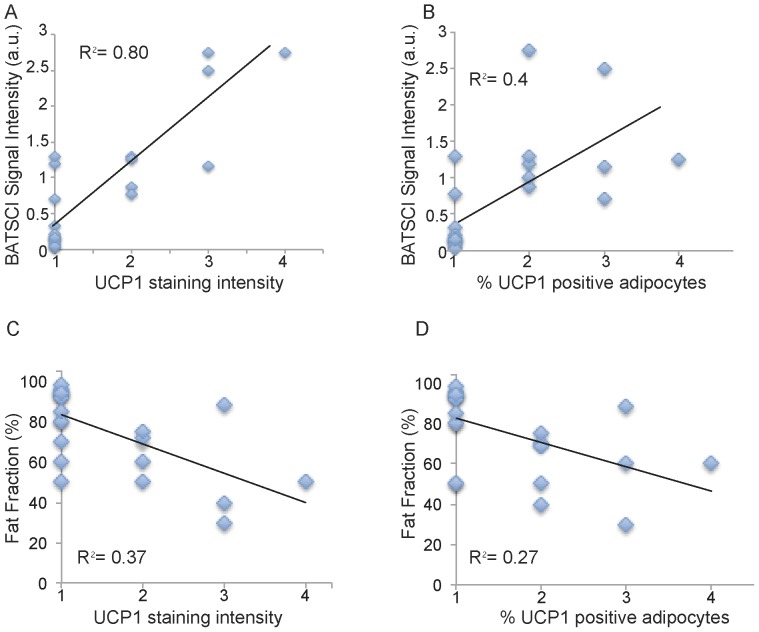
Correlation between BATSCI signal intensity and UCP1 expression. (**A**) Scatter plot showing the correlation between intensity of UCP1 staining and normalized BATSCI signal intensity (r-square = 0.8, p<0.0001). (**B**) Scatter plot showing correlation between % of adipocytes tissue stained positive for UCP1 and normalized BATSCI signal intensity (r square = 0.4, p<0.003). (**C**) Scatter plot showing the correlation between intensity of UCP1 staining and tissue fat fraction (r-square = 0.37, p<0.016). (**D**) Scatter plot showing correlation between % of adipocytes tissue stained positive for UCP1 and tissue fat fraction (r square = 0.27, p<0.0015).

Figure 7 shows BATSCI maps obtained from 3 of the 24 human tissue samples analyzed, specifically from the supraclavicular fat of a 28 year old male (I), and from the supraclavicular (II) and abdominal fat (III) of a 95 year old female. It is interesting to note that despite the similarity in overall fat fraction, as quantified by ^1^H-MRS spectra (D–E, inset), between the two supraclavicular samples (40±5% and 50±5% respectively), the relative ratio between the BATSCI signal and the nearby fat-fat signal (Fig.6 D–E) is very different. The supraclavicular sample (Fig. 7.L) from the young subject presents a large amount of UCP1 positive adipocytes (BAT cells), which leads to a large BATSCI signal (Fig. 7.B and 7.D) whose intensity is more than twice that of the nearby fat-fat iZQC peak (ratio >2). On the other hand, the sample excised from the older subject presents similar fat fraction but fewer brown adipocytes (Fig. 7.M) interspersed between normal white fat and connective tissue, thus leading to a BATSCI signal (Fig. 7.E and 7.B) whose intensity is greatly reduced with respect to the nearby fat-fat peak (ratio of 0.7). This ratio, rather than being the absolute intensity of the water-fat iZQC signal, seemed to be better correlated with the UCP1 staining intensity (Fig. 8A) while it was not correlated with the overall percentage of adipose tissue that stains positive for UCP1 (regardless of the staining intensity, Fig. 8B).

The intensity of the staining of brown adipocytes also varied greatly from subject to subject and also, in the same tissue, from cell to cell. Brown adipocytes, exhibiting multilocular cell morphology displayed strong immunoreactivity for UCP1, while brown adipocytes with an intermediate morphology between classical brown adipose tissue and white adipose tissue displayed a weaker immunoreactivity for UCP1. Concurrently, the ratio between the BATSCI signal and nearby fat-fat iZQC signal varied widely, between 0.01 and 3, assuming larger values for tissues excised from the supraclavicular area of younger cadavers, contained high amounts of UCP1-positive adipocytes with the classical brown fat morphology: adipocytes with a large number of small lipid droplets and high hydration. Lower ratio values were obtained for samples excised from the supraclavicular area of people showing mainly unilocular UCP1 positive adipocytes, i.e. brown fat cells that began to show a transformation into white fat cells, while values close to zero were obtained for all tissues excised from the abdominal cavity containing only UCP1-negative unilocular adipocytes.

A much lower correlation was found between UCP1 stain intensity and fat fraction measurements, while no correlation was found between fat fraction measurements and number of brown fat cells, as expected. The lower correlation found between UCP1 stain intensity and fat fraction measurements can be easily ascribed to the presence of other tissues in the samples analyzed. Specifically, supraclavicular samples were found to contain, in many cases, normal white fat as well as connective tissue. A large amount of connective tissue was also found histologically in the abdominal fat sample of one of the subjects, thus giving rise to a fat fraction similar to that of BAT.

### *In vivo* Detection of Human Brown Adipose Tissue

**Figure 9:**
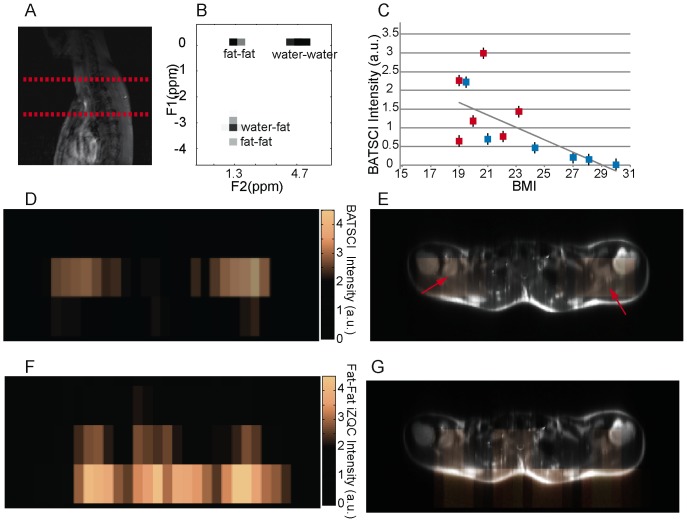
In vivo detection of human BAT in a lean subject. (**A**) 2D anatomical image showing the selected slice from which iZQC spectra and images were acquired. (**B**) 2D iZQC spectrum acquired from the neck-supraclavicular area of a young female with a BMI of 19. (**C**) Scatter plot showing the inverse correlation between subject BMI and normalized BATSCI signal intensity (r-value = 66%, r-square = 43%, p value less than 0.015). Blue solid squares indicate male subjects, while red solid squares indicate female subjects. (**D**) BATSCI map on the same subject. (**E**) Overlay of BATSCI on spin-echo axial image with red arrows indicating possible BAT locations. (**F**) Fat-fat iZQC map on the same subject. (**G**) Overlay of fat –fat iZQC map on spin-echo axial image.

The feasibility to use BATSCI to detect BAT in humans *in vivo* was evaluated using a commercial 3T clinical scanner (Tim-Trio, Siemens Healthcare, Erlangen, Germany). For these studies we enrolled 12 healthy volunteers with a BMI ranging from 19 to 28. For each subject we measured the intensity of the water-fat iZQC signal normalized to the intensity of the nearby fat-fat iZQC signal in a 20 cm slice covering the entire cervical–supraclavicular area and, in two cases, the abdominal area. Figure 9.B and 10.B show typical spectra acquired from the cervical–supraclavicular area of a lean and a normal weight subject, respectively. The spectrum acquired on the lean subject shows an intense water-fat iZQC peak at the expected water-fat iZQC resonance frequency (F1 = 4.7–1.3 = 3.4 ppm) nearby a fat-fat iZQC peak (F1 = 5.3–1.3 = 4 ppm). Contaminations at F1 = 0 ppm originating from both water-water and fat-fat iZQC signal can also be observed. Conversely, the spectrum from the normal weight subject shows a considerably reduced water-fat iZQC peak at the same location.

Figure 9.C shows the intensity of the BATSCI signal normalized to the intensity of the nearby fat-fat iZQC signal as function of the BMI for all subjects analyzed. A negative correlation seems to exist between the intensity of the BATSCI peak and the subject BMI (r = 0.66, P = 0.0035): all lean subjects had enhanced BATSCI signal with respect to overweight subjects. In addition a large span (0.5 to 3) in BATSCI signal intensity was also observed among the lean subjects.

**Figure 10:**
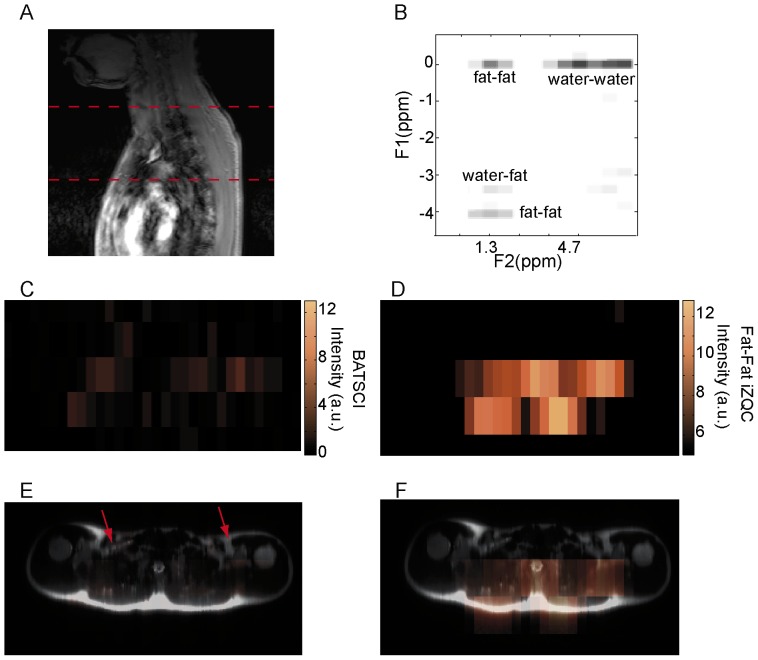
In vivo detection of human BAT in a normal weight subject. (**A**) 2D anatomical image showing the selected slice from which iZQC spectra and images were acquired. (**B**) 2D iZQC spectrum acquired from the same slice (neck/supraclavicular area) of a young male with a BMI of 24.5. (**C**) BATSCI map on the same subject. The BATSCI signal is undetectable and close to the noise level. (**D**) Fat-fat iZQC map on the same subject. The fat-fat iZQC map highlights mainly the subcutaneous fat layer. (**E**) Overlay of BATSCI on spin-echo axial image with red arrows indicating possible BAT locations. (**F**) Overlay of fat –fat iZQC map on spin-echo axial image.

Figure 9.D–G and figure 10.C–F show the BATSCI map and the fat-fat iZQC map obtained from the same subjects. In the lean subject the area with the most intense BATSCI signal correlates well with areas known to contain brown fat, although some positive BATSCI signal was clearly coming also from bone marrow, as expected. The fat-fat iZQC map, on the other hand, correlates well with the subcutaneous fat as well as with the supraclavicular fat depots. In the normal weight subject the BATSCI signal is close to the noise level, and no signal could be detected. The fat-fat iZQC maps, on the other hand, clearly highlight the subcutaneous depot, along with few other intra-muscle fat depots.

## Materials and Methods

### Ethics Statement

All human in vivo studies were approved by the Institutional Review Board of the University of North Carolina at Chapel Hill (IRB # 12–1229) and the University of Minnesota. All subjects gave written informed consent to the study.

All animals studies were performed with approval of the Duke University Institutional Animal Care and Use Committee (Registry Number: A085-11-04, Registry Title: In vivo Intermolecular Multiple Quantum Coherences).

Human tissue samples from cadavers were kindly donated by the Duke Human Fresh Tissue Laboratory (http://plastic.surgery.duke.edu/education-and-training/advanced-training/human-fresh-tissue-laboratory) and were exempt from IRB approval.

### Animal Studies

For the *in vitro* experiments excised tissues samples of interscapular BAT, visceral WAT and muscle were excised from 6 C57 6-week old male mice (Charles River).

For the *in vivo* imaging studies we used 14 C57 3-month old male mice, 4 nude mice, and 4 balb/c and 3 Cav1-mutated female mice at 12 weeks of age (Jackson Labs). Before the MR study, the animals were first induced with Nembutal (70 mg/kg) in a single intra-peritoneal injection. A catheter was then inserted in the intraperitoneal cavity to administer a maintenance dose of anesthesia (20 mg/Kg every 45 minutes or as needed). The animals were then placed in a supine position inside a birdcage ^1^H coil with a 35 mm inner diameter. Bore temperature was then monitored using MR-compatible temperature probes. At the end of the study, animals were euthanized through an overdose of pentobarbital. At the time of dissection the BAT areas were readily recognizable by gross inspection in the interscapular, axillary, and cervical portions of the anterior subcutaneous depots. They were dissected and fixed in formaline for histophatology evaluation.

All animals and *in vitro* MR studies were performed on a 7T small animal magnetic resonance tomograph with a 210 mm inner bore diameter, interfaced to a Bruker Biospec console (Bruker BioSpin MRI GmbH, Ettlingen, Germany). The system is equipped with a gradient coil system (maximum gradient strength 42 Gauss/cm) and a^ 1^H transmitter-receive coil with a 35 mm inner diameter.

For the *in vivo* imaging studies, coronal respiratory-gated spin echo images (TE = 14 ms, TR = 3 s, in plane resolution of 234 µm/pixel, slice thickness of 2 mm) were acquired.

The BATSCI maps were acquired with a modified version of the 2D iZQC spectroscopic sequence outlined in [28] to include read and phase encoding gradients. In the sequence, the first 90° pulse (Hermite, 1 ms duration) is used to excite water-fat double-quantum (iDQC) coherences. The frequency selective 180° pulse (Gaussian, 3.425 ms) applied at 1.3 ppm cross-converts water-fat iDQC into iZQC. A 90° broad-band pulse (Hermite, 1 ms) converts water-fat iZQC coherences into detectable single-quantum (iSQC) antiphase magnetization. This specific coherence transfer pathway (iDQC → iZQC → iSQC) is selected by a gradient combination of GT:G’T:2 GT = 8:−14∶16 Gauss * ms/cm. Finally, a slice selective refocusing pulse (Hermite, 1 ms), surrounded by crusher gradients, is applied to selectively refocus the signal from a single slice. A series of images (TR = 3 s, TE = 29 ms, NA = 4) were then collected by keeping the tau delay constant to 3.7 ms and by stepping the t1 delay (t1 = 3.4595 ms), during which time the water-fat iZQC signal evolves 32 or 64 times in increments of 0.333 ms. This results in a spectral bandwidth of 3000 Hz along the indirectly detected dimension F1 along which the iZQC peak is detected. The signal from each pixel of the 2D reconstructed images was then Fourier transformed along the t1 dimension to give rise to a 1D iZQC spectrum from each pixel. From this spectrum the BATSCI map was then reconstructed by measuring the area under the peak corresponding to the water-fat iZQC peak (at 3.4 ppm), while the fat-fat iZQC map was reconstructed by measuring the area under the peak corresponding to the fat-fat iZQC peak at 4 ppm.

### Fat Fraction Measurements

Fat fraction measurements were performed using frequency selective water and fat spin echo sequences (TE/TR = 11 ms/6 s, FOV = 60 mm*40 mm, MTX = 256*256, slice thickness = 2 mm) preceded by a saturation module (selective excitation by a 600 Hz Gaussian pulse followed by crash gradients along all 3 directions). More specifically, water images were acquired using a fat saturation suppression module followed by a water selective spin echo sequence. Similarly, fat images were acquired by using a water suppression saturation module followed by a fat selective spin echo sequence. Fat fraction images were then obtained using homemade Matlab code that performed a pixel by pixel calculation of fat fraction (SIfat/(SIfat+ SIwater)), where SIfat represents the fat image signal intensity and SIwater represents the water image signal intensity.

### *In vivo* Detection of BAT Activity

For the *in vivo* detection of BAT activity we used 6 C57 (Charles River) male mice. For these experiments we intubated and mechanically ventilated the animals at 60 breaths/min with a tidal volume of 0.3–0.4 ml and 21% O2, using an MR compatible ventilator. Two catheters were inserted in the intraperitoneal cavity, one to administer a maintenance dose of anesthesia (1/4 of initial dose every 40 minutes or as needed) and the other to administer norepinephrine (norepinephrine bitartrate monohydrate, Sigma-Aldrich, Saint Louis, Missouri, USA) (2.5 mg/kg) dissolved in 2 mM ascorbic acid solution in a single injection.

We detected BAT activity by collecting a series of 11 BATSCI images with very low spatial resolution (3.745 mm×3.745 mm×20 mm), every 15–30 minutes before, during and after stimulation of BAT. The acquisition of the BATSCI images was interleaved with the acquisition of high resolution T2* weighted images (TR = 3 s, Res = 0.3 mm×0.3 mm×1 mm/pixel) with a very short echo time (TE = 6 ms) to preserve signal intensity in BAT. We then evaluated the intensity of the BATSCI signal and the signal intensity of the T2* weighted images coming from interscapular brown fat region as function of time.

For the BAT temperature studies we used 6 C57 male mice and 6 balb/c (Charles River) female mice. After inducing anesthesia, we continuously monitored but did not maintain room, rectal, and interscapular BAT temperatures by the minute using fiber-optic temperature probes. For the placement of the interscapular BAT temperature probe, we made a surgical incision above the iBAT pad. The temperature probe was then slid underneath the iBAT pad and secured with 3 skin sutures and surgical tape. The rectal probe was inserted 1 cm beyond the anus and secured to the tail with surgical tape. Room temperature was measured with a temperature probe taped on the surgical pad next to the animal.

### ^18^F-FDG-PET Experiments

^18^F-FDG-PET experiments were performed on 2 C57 male mice within 32 hours from the MR study, using a dedicated Siemens micro-PET scanner for small animals with a reconstructed image resolution of 2 mm. For the ^18^F-FDG-PET scans, stimulation of BAT was induced by acute cold exposure (housing the animals at 4°C for 2 hours, in their cage with food and water ad libidum, before the injection of the radiotracer), and by leaving the animal in a cold bag of ice during radiotracer uptake. The animals were then transported to the imaging room for the injection of the radiotracer, which was given to the non-anesthetized animal in a single intra peritoneal injection of ^18^F-FDG (200 µCi in 0.2 ml). To prevent inhibition of BAT metabolic activity during ^18^F-FDG uptake the animals were kept conscious in their cages on a cold bag of ice and at a room temperature of 18°C. Before the scans the animals were anesthetized with a single I.P. injection of ketamine, which is known to stimulate the symphatetic nervous system [38]. PET scans were started 60 minutes after the injection of the radiotracer and lasted for about 40 minutes. PET images were then reconstructed using filtered back projection without scatter or attenuation correction.

### *In vitro* Human Studies

Human tissue samples were collected from 11 cadavers (6 male and 5 females) of age ranging between 28 and 95 years old. The brown adipose tissue was excised from the supraclavicular region, lateral to the sternocleidomastoid muscles and the edge of the posterior scalenus. The white adipose tissue was harvested from the subcutaneous region of the abdomen. Specimens were placed in a vial and analyzed with the BATSCI method as well as with conventional ^1^H MRS methods. At the end of the MR study, the tissues were fixed in 4% paraformaldehyde for 24 hours at 4°C, embedded in paraffin, and sectioned in 4 µm slices. The sections were de-waxed and rehydrated in a series of graded alcohols. Heat-induced epitope retrieval was performed in citrate buffer (pH 6.0) in a steamer for 20 minutes. The blocking procedure was carried out using Cyto-Q Background Buster (Innovex, USA; NB306) for 30 minutes followed by UCP1 primary antibody incubation for 1 hour at room temperature (1∶250; ab10983; Abcam, Cambridge, MA). Secondary antibody incubation and detection was performed for 30 minutes with biotinylated goat anti-rabbit (1;200; BA100; Vector, USA) and Vectrstain Elite ABC complex respectively (Vector, USA). The slides were then incubated in diaminobenzidine and counterstained in hematoxylin. Brown adipose tissue from a mouse was used as a positive control. Normal rabbit serum was used as a negative control.

### Histological Evaluation and Statistical Analysis

Sections were evaluated for abundance of UCP1-positive adipocytes and for UCP1 stain intensity by two pathologists, blinded to tissue group identity and MR results. Tissue scoring scale for both the adipose cells expressing the antibody and the intensity of the stain was: none/minimal = 1, mild = 2, moderate = 3, marked = 4. Simple linear regression analysis was performed on the *in vitro* human tissue data to identify correlation between UCP1 expression and BATSCI signal intensity.

### *In vivo* Human Studies

*In vivo* human studies were performed on two 3-T horizontal bore magnet (Tim-Trio Siemens). Both systems were equipped with a body coil for signal transmission and a spinal array coil for signal reception. The spinal array coil was located such that the entire neck-supraclavicular area was covered by the receiver. No respiratory gating was applied to the sequence. Before the iZQC experiment a localization experiment was performed to position the area of interest at the iso-center. After the localization experiment a B1 mapping was used to accurately calibrate the RF pulses in the area of interest. For these experiments both 2D-iZQC spectra and 3D-iZQC data sets for iZQC water-fat signal mapping were acquired. For these experiments the spectroscopy sequence was slightly modified to reduce contaminations coming from imperfect spin excitation. Specifically, all excitation pulses were substituted with adiabatic bir4-pulses (5.12 ms duration). For the acquisition of the 2D spectrum the t1 delay (8.62 ms) was then stepped 32 times in increments of 900 microseconds. Each scan was acquired with a repetition time of 3.2 s a number of averages of 8, resulting in a total scan time of about 15 minutes. For the acquisition of the BATSCI maps a total of 16 images (TR = 3 s, BW = 390 Hz/pixel, 8 averages, Res = 15×62×100 mm/pixel) were acquired with different values of t1 for a total scan time of 50 minutes.

## Discussion

This study describes and evaluates the use of a novel, noninvasive MRI method named BATSCI to image BAT mass and function in both mice and humans. The main hypothesis is that BATSCI signal originates exclusively from BAT and can therefore be used to map this tissue in the body. This is because BATSCI signal comes primarily from correlations between water and fat spins separated by a user-controlled distance, the “correlation distance”. By choosing a correlation distance less than 100 microns, we can probe the relative concentration of water and fat spins at a this “cellular level”, a scale which goes far beyond that commonly accessible by standard magnetic imaging methods. This eliminates confounding partial volume effects that make conventional fat fraction MR measurements, currently under investigation for the detection of BAT in humans, less specific.

This study builds upon our previous spectroscopy studies on BAT [28], in which a strong positive association was found between BATSCI signal intensity and BAT mass. The *in vivo* imaging experiments performed here, along with the histological and ^18^F-FDG-PET evaluations, allow us to conclude that the BATSCI signal originates, at least in rodents, from BAT. Therefore, in these animals, BATSCI can be used to identify and map BAT. Our animal experiments clearly show that BATSCI identifies the major BAT locations (the intrascapular and the peri-renal depots). In addition, BATSCI maps are very similar to the BAT maps obtained by ^18^F-FDG-PET scans, and correlate well with BAT maps obtained with conventional fat-fraction MR measurement methods, despite the very different image resolution used for these scans.

In addition, with our *in vivo* mice studies we show the possibility to detect BAT activity. BATSCI signal is very sensitive to the local changes in blood oxygenation that occur at the cellular level during BAT activity, suggesting a new noninvasive way to investigate BAT function. At the same time, the strong local change in susceptibility and intrinsic low BAT T2* values [39] (in some cases <5 ms) prevent the possibility to obtain reliable and accurate temperature maps [40], for which a significant signal evolution during the indirect t1 dimension is required.

This study also reports on the feasibility to conduct these studies in humans. Unlike in mice, in humans the association between BATSCI and BAT cannot be uniquely established without guided biopsy or combined ^18^F-FDG-PET scans, although the enhanced intensity of the BATSCI signal in those areas that are known to contain BAT is encouraging. Moreover, the strong positive correlation found between BAT mass and BATSCI signal intensity in the *in vitro* experiments on human brown adipose tissue also suggests the possibility to use the BATSCI signal to uniquely identify BAT in humans. In these *in vitro* experiments we observed a strong positive association between BATSCI signal intensity and UCP1 staining intensity, but at the same time an absence of correlation between BATSCI signal intensity and number of BAT cells. This apparent discrepancy is not surprising and simply reflects the expected variation in tissue fat content [41]. Multilocular brown fat cells (higher UCP1 stain intensity) with a mean fat fraction close to 50% always lead to a higher BATSCI signal than lipid rich brown fat cells (lower UCP1 staining intensity), simply because a larger number of possible correlations exists between water and fat spins in these cells. The intensity of the BATSCI signal depends, indeed, on to the relative concentration and distribution of water and fat spins at a distance of about the correlation distance, with the maximum signal expected for a fat fraction close to 50% and a homogeneous fat distribution in the cell. A reduced sensitivity is, on the other hand, expected for hyperactive (lipid depleted) or hypoactive (lipid rich) BAT, both of which are not expected to give an observable BATSCI signal. This reduced sensitivity of BATSCI to more lipid rich BAT cells could also explain the inability to collect a BATSCI map in some of the young normal weight subjects analyzed. Although for lipid rich BAT cells one further increases the selected correlation distance to account for the increased water - fat spin mean separation in the brown fat cell, in practice, contamination from coherences between water and fat spins that reside in different tissues, as well as contamination from other coherences that are not filtered out by the weakened correlation gradients, may become a serious concern.

Undoubtedly, the most significant advantage of this methodology with respect to conventional fat fraction measurements is its immunity to partial volume effects. As the *in vitro* human studies have shown, samples with similar fat fraction can give rise to completely different BATSCI signal intensities, whose strength depends exclusively on the water-fat distribution at the cellular level, and therefore, on their cellular composition. This extreme sensitivity of BATSCI to BAT cellular composition was observed in *in vitro* studies of human BAT, and more likely was the major cause of the large variation in BATSCI signal intensity found in our *in vivo* human studies. In this small study we didn’t find any increased incidence of BAT in females versus males. On the other hand, consistent with the previously observed inverse correlation between BMI and BAT mass, we noticed a negative correlation between the intensity of the BATSCI peak and the subject BMI. Clearly this correlation cannot entirely be attributed to an enhanced BAT content, or to the presence of BAT with the classical morphology (50% fat, 50% water), since a lower white fat content easily leads to a lower fat-fat iZQC peak and a higher normalized BATSCI signal.

Aside from its immunity to partial volume effects, it is important to point out that this methodology presents the same limitations of other fat fraction measurement methods that rely on the peculiar water-fat content of BAT for detection. Knowing that in BAT water-fat composition can vary substantially from a nearly fat depleted stage to a more lipid-full stage [13], [41], where lipid vacuoles start to coalesce in a single large lipid droplet, we expect these methodologies to have limited sensitivity for the detection of human BAT in very lean subjects or, more importantly, in overweight or obese subjects for which these detection methods are more needed. To overcome this limitation, this author is concurrently investigating other proton [36] and non-proton MR methodologies, like 129Xe-MRI, that do not base their detection on the peculiar fat composition of BAT but are more sensitive to those physiological changes associated with BAT activity (blood flow, temperature).

BATSCI method is a research tool that still needs extensive technical improvements before it can be widely utilized in clinical work. Currently the major limitation is the very low SNR of the BATSCI images. Because of the intrinsically low T2 and T2* of this tissue, which limit the use of long sequence delays needed for BATSCI signal growth [30], the BATSCI in BAT is only a very small fraction (<10%) of the conventional MR signal. This requires the use of extensive signal averaging to boost the BATSCI signal and to reduce contaminations from other coherences. In addition, the CSI-type acquisition scheme adopted here further increases the acquisition time necessary to obtain reasonable-looking images. In this respect, imaging acquisition speed could be greatly reduced to a more manageable duration by completely suppressing, through a clever design of the pulse sequence, all possible signal contaminations that come from fat-fat and water-water iZQC coherences, leaving only BATSCI signal for detection. Another possibility would be to use localized BATSCI spectroscopy to estimate BAT incident in the human population, or in conjunction with fat-fraction measurements [39], [42] in studies aiming to identify BAT more accurately.

The present study did not conclusively show that in humans, as in mice, BATSCI maps correspond to BAT maps. A major limitation of our *in vivo* human imaging study is the lack of a validation or comparison with conventional ^18^F-FDG-PET scans or fat-fraction imaging methods that are gaining popularity for the detection of BAT. This comparison was not done because of the already long imaging examination, which overall was already close to 2 hours. In addition, before these comparisons can be made, the BATSCI image resolution should be considerably improved. Because of the very different image resolution between BATSCI and fat fraction measurements (especially along the slice direction) and because of the very diffuse distribution of BAT in humans, such comparison is not expected to be as straightforward as in mice.

Our studies only suggest the possibility to use BATSCI to detect human BAT but clearly more experiments are needed to validate this methodology in humans. Further investigations should also be considered to assess its sensitivity to different “shades” of BAT, as well as the extent of possible contaminations from intramyocellular lipid or other tissues that closely resemble BAT structures, like bone marrow.

Despite these limitations, the possibility to detect BAT with MR, independently from its activity, is an important feature that will inherently lead to fewer false negative results than ^18^F-FDG-PET scans. This will facilitate future studies in which we aim to determine the true prevalence of this tissue in the adult human population and to detect its metabolic dysfunction.
